# Most respiratory symptoms have resolved 9 years after PM_2.5_ exposure from the Hazelwood coal mine fire

**DOI:** 10.1097/EE9.0000000000000450

**Published:** 2025-12-30

**Authors:** Catherine L. Smith, Caroline X. Gao, Claire F. O’Sullivan, Brigitte M. Borg, Tyler J. Lane, David Brown, Jillian Ikin, Matthew T.C. Carroll, Karen Walker-Bone, Bruce R. Thompson, Michael J. Abramson

**Affiliations:** a School of Public Health and Preventive Medicine, Monash University, Melbourne, Victoria, Australia; b Orygen, Centre for Youth Mental Health, The University of Melbourne, Victoria, Australia; c Respiratory Medicine, The Alfred, Victoria, Australia; d Monash Rural Health Churchill, Monash University, Victoria, Australia; e School of Health Sciences, University of Melbourne, Parkville, Australia

**Keywords:** Air pollution, Respiratory symptoms, Coal mine fire smoke, PM_2.5_

## Abstract

**Background:**

In 2014, a coal mine fire in regional Australia exposed the local community to 6 weeks of hazardous fine particulate matter less than 2.5 µm in diameter (PM_2.5_). We investigated longitudinal associations between PM_2.5_ exposure and respiratory symptoms over 9 years postfire.

**Methods:**

In 2016–2017 (wave 1, W1), 4,056 exposed and unexposed adult residents completed surveys, including validated questionnaires for respiratory symptoms. Individual PM_2.5_ exposures were estimated using modeled smoke data and time-location diaries. Respiratory symptoms were followed up 4-, 7-, and 9 years postmine fire (W2, W3, and W4) in a weighted random subsample of 519 participants (346 exposed) oversampled for people with asthma. Longitudinal associations between mine fire PM_2.5_ and respiratory symptoms were assessed using mixed-effects logistic regression, adjusting for age, sex, socioeconomic factors, smoking, occupational exposures, and self-reported chronic respiratory diseases diagnosed premine fire.

**Results:**

Several patterns were observed. PM_2.5_ exposure was associated with elevated wheeze across the survey waves, and the associations tended to be higher among participants with premine fire asthma. Dyspnea (nocturnal and resting) increased at the first survey wave but was not detectable afterwards. Chest tightness and chronic cough had detectable increases at follow-up (W2 for both, W3 for chest tightness), but attenuated in the following surveys. No effects were detectable for chronic phlegm or nasal symptoms.

**Conclusion:**

While there was some variation over time, exposure to mine fire-related PM_2.5_ was no longer associated with chest tightness, dyspnea, and chronic cough 9 years postmine fire. The exception was wheeze, where effects of PM_2.5_ exposure persisted 9 years later, particularly in people with asthma.

**What this study adds**

This study provides valuable insights into the long-term respiratory impacts of exposure to coal mine fire smoke, specifically focusing on the effects of exposure to fine particulate matter less than 2.5 µm in diameter on respiratory symptoms and how they change over time. It extends previous research by examining data from surveys conducted at four separate time points, offering a clearer understanding of symptom trajectories. Oversampling for people with asthma, a potentially vulnerable group, adds depth to the findings, revealing that certain respiratory symptoms, like wheeze, remain associated with exposure to fine particulate matter less than 2.5 µm in diameter in the long term. These findings underscore the need for continued monitoring and targeted interventions for affected communities.

## Introduction

Coal mine fires can burn for extended periods^1^ and are likely to become more prevalent as climate change contributes to increasingly frequent and severe wildfires.^2,3^ Understanding both the short- and long-term health consequences of exposure to smoke from these fires is crucial, particularly for affected communities, to better prepare for a future with more frequent smoke events. We focused on long-term impacts, specifically the burden of respiratory symptoms.

Although direct evidence linking coal mine fires to long-term respiratory outcomes is limited, the smoke they emit shares a similar profile to wildfire smoke.^4^ Both contain fine particulate matter less than 2.5 µm in diameter (PM_2.5_), small enough to penetrate deep into the lungs.^5^ Wildfire smoke is well-documented to cause short-term respiratory harm in vulnerable populations, such as individuals with preexisting cardiac or respiratory conditions.^4,6^ Short-term (hours to days) exposure to PM_2.5_ has been estimated to cause around one million premature deaths globally each year.^7^ Long-term effects of wildfire smoke on respiratory health are less certain. A recent systematic review found limited evidence of long-term respiratory morbidity associated with wildfire smoke, but acknowledged the need for further research, including studies targeting vulnerable populations.^8^

Much of the existing evidence on the respiratory health impacts of coal mine fires comes from the Hazelwood Health Study (HHS), an independent research program funded by the Victorian Government in response to extreme PM_2.5_ exposure experienced by residents of the Latrobe Valley, south-eastern Australia. This exposure was related to a 6-week coal mine fire in February 2014, ignited by embers from nearby wildfires. During the event, which became known as the Hazelwood mine fire, modeled PM_2.5_ concentrations reached extreme levels, with hourly PM_2.5_ peaking at 3,700 µg/m³, and daily average levels peaking at 1,022 µg/m³, repeatedly exceeding the daily average limit of 25 µg/m³ set by the National Environment Protection Measures.^9^ During and after the event, residents of the nearby affected community reported numerous symptoms they attributed to the mine fire and raised concerns about potential long-term health effects.^10^ In response, the HHS was launched as a comprehensive, multi-stream, 10-year research program aimed at investigating the health effects associated with the fire.

Results to date have identified several short-term risks associated with mine fire-related PM_2.5_ exposure, including increased emergency department (ED) presentations and hospital admissions for respiratory diseases such as asthma and chronic obstructive pulmonary disease (COPD) during and immediately after the fire.^11^(11, 12) There was also an increase in the dispensing of respiratory medications^12^ and use of respiratory health services.^13^ The Adult Survey, conducted in 2016–2017, enrolled residents from Morwell (the most heavily exposed area during the mine fire) and selected areas of Sale (a nearby unexposed town) with similar demographic and socioeconomic characteristics, establishing a cohort for ongoing investigations.^14^ Analyses of the survey data have found increased risks of self-reported chronic cough, phlegm, and wheeze in association with increasing mine fire-related PM_2.5_ exposure, 2.5 years after the mine fire.^15^ Administrative health data linked to the cohort showed increases in respiratory-related ambulance attendances at 3.5 years^16^ and increased respiratory ED presentations^17^ and hospitalizations^18^ 5 years postmine fire. A single follow-up 8.5–9 years postfire showed a worsening of symptoms of chronic cough and potentially current wheeze, in association with increasing PM_2.5_.^19^

To better understand the mine fire’s impact on respiratory health trajectories—particularly among individuals with preexisting conditions—the Respiratory Stream of the HHS recruited a subsample of the Adult Survey cohort for comprehensive respiratory function testing and symptom assessment at three further time points. The Respiratory Stream oversampled individuals with asthma, known to be particularly vulnerable to the acute effects of wildfire smoke.^20^ Longitudinal lung function findings in this cohort suggested a worsening of lung mechanics 4 years postfire,^21^ with improvements observed at 7 years ^22^ and 9 years^23^ postfire, suggesting lung function recovery. The Respiratory Stream also observed a dose-response association between mine fire-related PM_2.5_ exposure and spirometry consistent with COPD in nonsmokers, 4 years after the fire.^24^

The current analysis focuses on four rounds of respiratory symptoms reported by the Respiratory Stream subcohort, incorporating data from the Adult Survey and the three subsequent follow-up time points. By assessing symptoms at multiple time points, the analysis aims to provide detailed estimates of the long-term effects of fire-related PM_2.5_ on symptom trajectories in a group oversampled for preexisting asthma. This work complements and expands upon previous findings at two time points in a group that was not oversampled for asthma.^19^

## Methods

### Study design and setting

Details of the Adult Survey cohort have been published elsewhere.^14^ In brief, 3,096 adults from Morwell and 960 from Sale were surveyed between May 2016 and February 2017 (wave 1; W1). The Respiratory Stream recruited 519 people (the respiratory subcohort; 346 from Morwell and 173 from Sale) from a weighted random sample of 1,346 Adult Survey participants, with oversampling of self-reported premine fire asthma in both towns comprising around 45% of the final sample. The respiratory subcohort was invited to undertake clinical assessments and to complete respiratory symptom surveys between August 2017 and March 2018 (wave 2; W2), May to November 2021 (wave 3; W3), and June to October 2023 (wave 4; W4).

### Respiratory symptoms

In all four waves of data collection, self-reported respiratory symptom questions were derived from a modified version of the European Community Respiratory Health Survey Short Screening Questionnaire^25^ and included: current wheeze; chest tightness; nocturnal dyspnea; dyspnea while resting; nasal symptoms without having a cold, all in the previous year; chronic cough and chronic phlegm, in at least three out of the previous 12 months.

### Mine fire PM_2.5_ exposure assessment

Individual-level mean daily mine fire-related PM_2.5_ exposure was calculated by mapping time-location diaries recorded in the Adult Survey onto hourly spatial estimates of mine fire-related PM_2.5_ exposure emissions during the mine fire.^15^ Due to the absence of ground-level monitoring during the first few days of the fire, spatial estimates were modeled by the Commonwealth Scientific and Industrial Research Organization using emissions and chemical transport modeling, which accounted for meteorological conditions at the time.^9^

### Statistical analysis

Characteristics of the respiratory stream participants at W1 were summarized by exposure group using descriptive statistics. Continuous variables were reported as medians with interquartile ranges (IQR) and categorical variables as frequencies and percentages. Comparisons of categorical variables were conducted using Fisher’s exact tests, while continuous variables were compared using Wilcoxon rank-sum tests. The proportions of respiratory symptoms over time, stratified by exposure group, are presented as bar charts.

Mixed-effects logistic regression was used to model the association between individual-level PM_2.5_ exposure and respiratory symptoms over time. PM_2.5_ was treated as a continuous variable, while time was modeled categorically, with W1 as the reference group. An interaction term between time and PM_2.5_ was included in all models to estimate the PM_2.5_ exposure effect at each survey wave, using linear combinations of the regression coefficients. The effect of PM_2.5_ exposure was expressed as an odds ratio (OR) with a 95% confidence interval (CI), representing the multiplicative change in the odds of reporting a given symptom for every 10 µg/m^3^ increase in mine fire-related PM_2.5_ exposure.

All models were adjusted for confounders self-reported at W1: age, sex, education (postsecondary vs secondary), employment status (in paid employment vs not), smoking status (never, former, and current smoker), cigarette pack years (log transformed after adding 1), occupational exposure, defined as at least 6 months in a job involving dust, fumes, smoke, gas vapor or mist, including in a coal mine, power station, or emergency service, and a combined indicator of either self-reported doctor-diagnosed premine fire asthma or COPD. To account for socioeconomic status, we also adjusted for the Index of Relative Socioeconomic Advantage and Disadvantage based on 2011 Australian Census data.^26^ Index of Relative Socioeconomic Advantage and Disadvantage provides a summary measure of relative advantage (e.g., higher income and skilled occupations) and disadvantage (e.g., low income and unemployment) in a given geographical area, with higher scores indicating greater levels of advantage.

Multiple imputation was used to address missing data, including outcomes for participants lost to follow-up at W3 and W4.^27^ The data were imputed in wide format using random forest multiple imputation via the missRanger package^28^ in R software^29^and reshaped long for analysis. Fifty imputed datasets were created for regression analyses, and results were combined using Rubin’s rules.^30^ A secondary analysis was conducted, stratifying by premine fire asthma status. All analyses were conducted using R Software in RStudio.^31^

### Sensitivity analyses

We conducted two sensitivity analyses. The first included the same imputed data used in the primary analysis, but incorporated inverse probability weighting in the modeling to adjust for the oversampling of individuals with asthma. The second analysis was restricted to participants observed at each survey wave: 519 in W1 and W2, 341 in W3, and 244 in W4 (not imputing for outcomes due to loss of follow-up). For these participants, any missing data were addressed using multiple imputation. To assess potential bias due to attrition, we conducted a descriptive analysis of W4 responders (n = 244) and nonresponders (n = 275) to compare characteristics and the respiratory symptoms in W1–W3.

This study was approved by the Monash University Human Research Ethics Committee (project numbers 6066, 25680, and 36471) and the Alfred Hospital Ethics Committee (project number 90/21). All participants provided written informed consent.

## Results

### Participants

The Respiratory Stream cohort included 519 participants, representing 39% of the 1,346 invited from the Adult Survey cohort. Compared with nonparticipants, those who participated were older, less likely to be in paid employment, and less likely to be current smokers.^21^ Of the 519 Adult Survey (W1) members who formed the respiratory subcohort (W2), 341 (227 exposed) subsequently participated at W3 and 244 (164 exposed) at W4. Details of losses to follow-up are given in Figure S1; https://links.lww.com/EE/A396. Participant characteristics of the respiratory subcohort at W1 are provided in Table 1. Age, sex, education level, occupational exposure, and smoking were comparable between the two exposure groups. However, compared with Sale, Morwell had a greater relative disadvantage and twice the proportion of participants unemployed or unable to work. A total of 232 participants self-reported a premine fire asthma diagnosis, slightly more common in Morwell than in Sale. Of these, 14 participants with asthma also self-reported a premine fire diagnosis of COPD. Participant characteristics in W3 and W4 are provided in Table S1; https://links.lww.com/EE/A396.

**Table 1.** Participant characteristics within exposure groups, in Wave 1

	Morwell (exposed)	Sale (unexposed)	
	N = 346	N = 173	*P* value
Daily mean mine fire-related PM_2.5_ µg/m^3^, median (IQR)	11.7 (7.2, 18.4)	0.0 (0.0, 0.0)	
Age in years, median (IQR)	53.5 (40.0, 65.0)	55.0 (43.0, 66.0)	0.284
Female, n (%)	195 (56.4%)	111 (64.2%)	0.107
Education, n (%)			
Up to year 10	85 (24.9%)	34 (19.8%)	0.208
Year 11–12	72 (21.1%)	31 (18.0%)	
Postsecondary	184 (54.0%)	107 (62.2%)	
Employment status, n (%)			
Employed	154 (44.8%)	88 (51.8%)	0.021
Other (retired, home, study)	132 (38.4%)	68 (40.0%)	
Unemployed/unable to work	58 (16.9%)	14 (8.2%)	
Occupational exposures, n (%)	141 (40.8%)	64 (37.0%)	0.446
IRSAD score (2011), median (IQR)	854 (806, 892)	900 (858, 962)	<0.001
Self-reported premine fire asthma, n (%)	165 (47.8%)	67 (39.0%)	0.061
Self-reported premine fire COPD, n (%)	16 (4.6%)	5 (2.9%)	0.479
Smoking status, n (%)			
Never-smoker	175 (50.6%)	89 (51.4%)	0.516
Ex-smoker	114 (32.9%)	62 (35.8%)	
Current smoker	57 (16.5%)	22 (12.7%)	
Cigarette pack years in smokers, median (IQR)	13.5 (4.7, 25.0)	15.0 (5.8, 29.5)	0.671

Missing data ranged from 0% to 2.3%.

IRSAD indicates index of relative socio-economic advantage and disadvantage.

### Respiratory symptoms over time

Figure S2; https://links.lww.com/EE/A396 shows the proportions of participants reporting each respiratory symptom in each town by survey wave. With the exception of nasal symptoms, other respiratory symptoms in Morwell tended to decrease across the waves, yet generally remained higher than in Sale. The prevalence of nasal symptoms was fairly steady in both towns, with a small decline after W1 observed in Morwell.

### The effects of PM_2.5_ exposure

**Figure 1.**
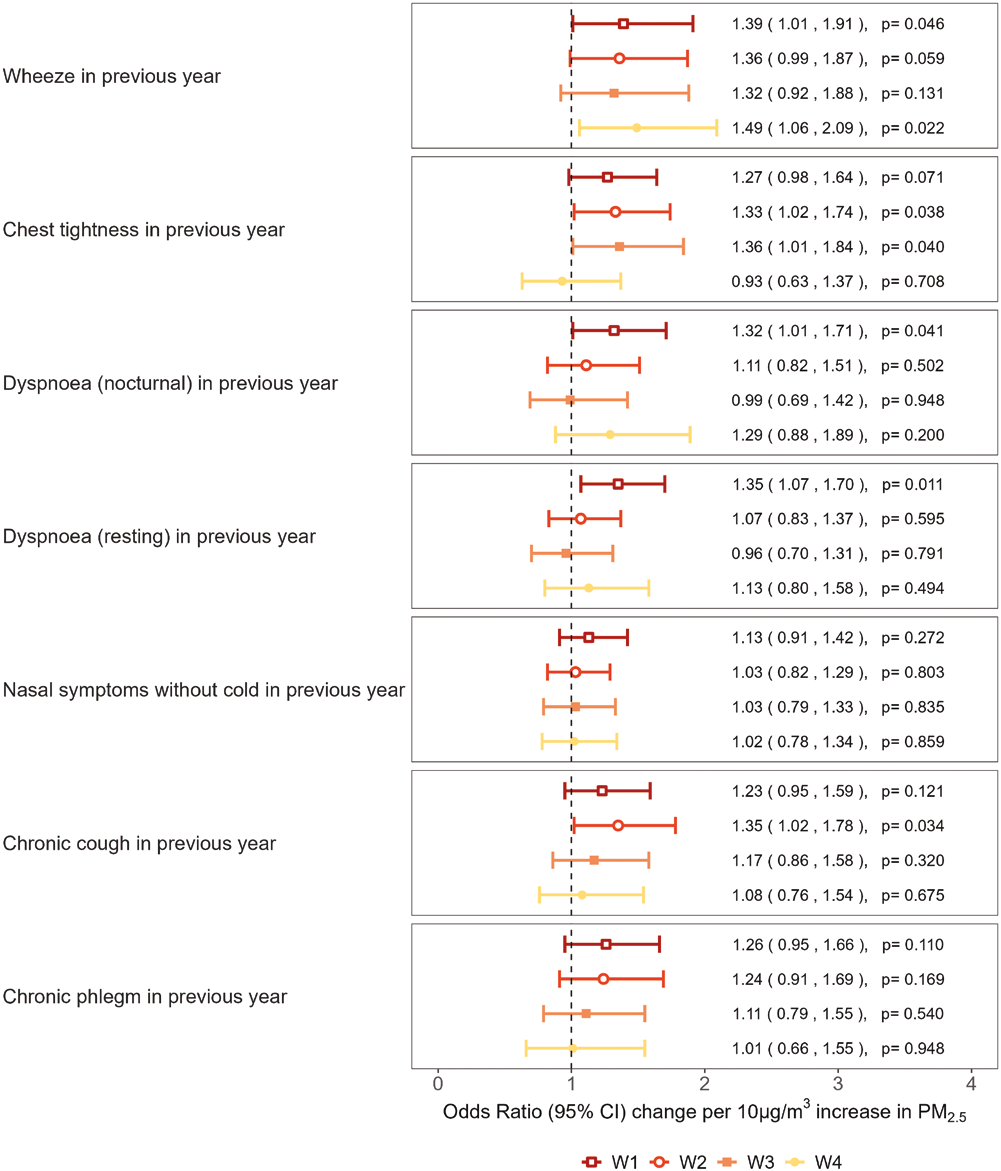
Associations between PM_2.5_ and respiratory symptoms at each wave. Notes: “Chronic” is defined as at least 3 of the previous 12 months. All models were adjusted for age, sex, employment status, education level, socioeconomic status, smoking, cigarette pack years, occupational exposure, and self-reported asthma or COPD diagnosed premine fire.

Figure 1 shows the estimated associations between mine fire-related PM_2.5_ exposure and respiratory symptoms at each survey wave. A pattern of increasing odds of wheeze with increasing mine fire PM_2.5_ exposure was observed across all survey waves, significantly at W1 and W4. For every 10 µg/m^3^ increase in exposure, the estimated odds of wheeze increased by 39% in W1 (OR = 1.39, 95% CI = 1.01, 1.91), 36% in W2 (OR = 1.36, 95% CI = 0.99, 1.87), 32% in W3 (OR = 1.32, 95% CI = 0.92, 1.88), and 49% in W4 (OR = 1.49, 95% CI = 1.06, 2.09). The odds of chest tightness increased with PM_2.5_ exposure in the first three waves, reaching an OR of 1.36 (95% CI = 1.01, 1.84) in W3 before attenuating to a null effect in W4.

The patterns of association with PM_2.5_ were similar for both nocturnal and resting dyspnea, with increased odds only in W1 (OR = 1.32, 95% CI = 1.01, 1.71 for nocturnal; OR = 1.35, 95% CI = 1.07, 1.71 for resting). For chronic cough, the highest risk was observed in W2, with a 35% increase in odds per 10 µg/m³ PM_2.5_ (OR = 1.35, 95% CI = 1.02, 1.78); no effect was evident in W3 or W4. The OR for chronic phlegm was highest in W1 (OR = 1.26, 95% CI = 0.95, 1.66), decreasing in subsequent waves, although all estimates were consistent with no effect. No associations were detected for nasal symptoms, and none of the interaction tests between PM_2.5_ exposure and survey wave were statistically significant.

**Figure 2.**
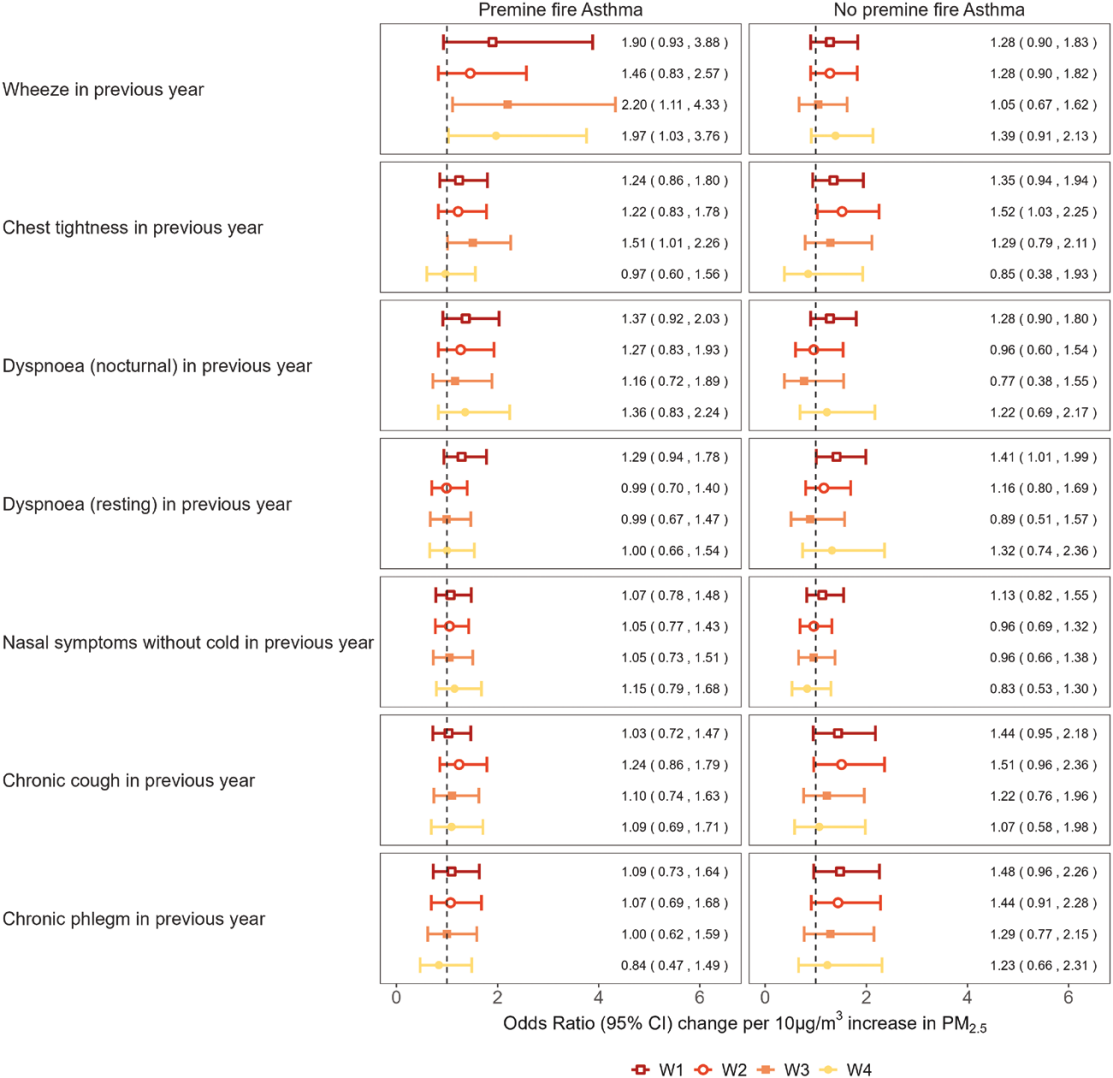
Associations between PM_2.5_ and respiratory symptoms at each wave, stratified by premine fire asthma status. Notes: “Chronic” is defined as at least 3 of the previous 12 months. All models were adjusted for age, sex, employment status, education level, socioeconomic status, smoking, cigarette pack years, and occupational exposure.

The results of the stratified analyses are presented in Figure 2. Across the survey waves, the odds of wheeze associated with PM_2.5_ exposure tended to be higher among participants with premine fire asthma compared to those without. Among participants without premine fire asthma, the estimated ORs for waves 1, 2, and 4 were greater than one, although the corresponding CIs included the null value. In contrast, for chest tightness and chronic cough, stronger associations with PM_2.5_ during the first two survey waves were observed in participants without premine fire asthma. The ORs for dyspnea were similar between those with or without preexisting asthma.

### Sensitivity analyses

The results of the primary analysis were compared with the inverse probability weighted analysis in Figure S3; https://links.lww.com/EE/A396, and with the observed participant data analysis in Figure S4; https://links.lww.com/EE/A396. Although the weighted estimates had wider CIs, the direction of the estimates and conclusions were consistent with the unweighted results (Figure S3; https://links.lww.com/EE/A396). Similarly, Figure S4; https://links.lww.com/EE/A396, shows that the primary analysis results were very similar to the results had we not imputed data for those lost to follow-up in W3 and W4. This finding is consistent with the descriptive comparison of W4 responders and nonresponders presented in Table S2; https://links.lww.com/EE/A396, which showed no evidence that respiratory symptoms across W1–W3 varied according to participation in W4.

## Discussion

In this analysis, we investigated the long-term, longitudinal impact of 6 weeks of exposure to coal mine fire-related PM_2.5_ on respiratory symptoms in a cohort oversampled for people with asthma. We found evidence of a persistent effect of PM_2.5_ exposure on wheeze, lasting up to 9 years after the fire, particularly in those with premine fire asthma. In contrast, associations between PM_2.5_ exposure and other symptoms—chest tightness, chronic cough, and dyspnea (nocturnal and resting)—were no longer evident by the final survey wave, although the timing of attenuation varied by symptom. Effects on chest tightness persisted up to 7 years postfire, while effects on chronic cough were most pronounced 4 years postfire. For both nocturnal and resting dyspnea, increased risk was observed only at 2.5 years after the fire. PM_2.5_ exposure was not associated with nasal symptoms or chronic phlegm at any survey wave.

A consistent finding across this study and our previous HHS analysis^19^ was the persistent effect of mine fire-related PM_2.5_ exposure on increased wheeze 9 years postfire, with this increase observed consistently across all four time points. The earlier HHS analysis,^19^ was based on an Adult Survey subcohort not oversampled for asthma, and persistent effects on wheeze were predominantly found among people without premine fire asthma. In contrast, in our respiratory subcohort, the persistent effects on wheeze were greatest in participants with premine fire asthma. These differences likely reflect the oversampling of people with asthma in the current study. Almost half of our participants (232/519) were people with premine fire asthma compared with one quarter in the previous study sample (160/612), potentially giving this study greater power to detect any effects in those with premine fire asthma, but less power to detect effects among those without.

Wheeze is a symptom of airway closure or obstruction and is commonly experienced by individuals with asthma. The persistence of wheeze associated with mine fire-related PM_2.5_ exposure may reflect several overlapping mechanisms. For example, wheeze could indicate uncontrolled asthma, as found in an initial assessment of people with asthma in the respiratory subcohort at W2.^32^ PM_2.5_ exposure may lead to episodic bronchoconstriction through increased airway inflammation and subsequent increased bronchomotor tone, consistent with evidence from a nation-wide Danish case–control study showing that children exposed to higher levels of ambient PM_2.5_ were more likely to develop asthma and persistent wheezing.^33^

In our cohort, the persistent wheeze may also reflect long-term airway changes following PM₂.₅ exposure. A review by Xing et al^34^ found that the ability of PM_2.5_ to reach the lung periphery can result in alveolar wall damage and impaired lung function. Further, an examination of normal lung tissue found incomplete clearance of deposited urban sources of PM_2.5_.^35^ The resultant irritation and/or remodeling of the small airways may increase the propensity for airway closure on expiration, and may contribute to persistent wheeze years after exposure in this cohort. This theory is in keeping with our previous findings, where lung function testing in this study cohort showed an association between mine fire-related PM_2.5_ exposure and increased heterogeneity of ventilation, with poorer gas mixing in the conductive airways.^36^

Importantly, PM_2.5_ from coal mine fires may be even more harmful than urban PM_2.5_, as it has a chemical profile similar to wildfire PM_2.5_,^4^ which contains a greater fraction of submicronic and ultrafine particles^6^ and higher concentrations of toxic components.^37^ These components include a mixture of organic compounds (such as polycyclic aromatic hydrocarbons) and inorganic substances (such as metals and other toxic compounds), which contribute to greater oxidative stress than urban PM_2.5_.^38^ Aguilera et al^39^ found that PM_2.5_ from wildfire smoke had a bigger effect on respiratory hospitalizations than nonwildfire PM_2.5_. In our cohort, exposure to smoke from the 2019 to 2020 Black Summer bushfires in Australia, which were unprecedented in scale and duration,^40^ may have further contributed to persistent wheeze in our participants, potentially compounding the long-term effects of the earlier coal mine fire. In Victoria alone, smoke from these fires was associated with 401 asthma-related ED attendances and 585 respiratory-related hospital admissions during that period.^40^

Beyond biological effects, health-related behaviors such as medication adherence or healthcare access may have influenced the persistence of wheeze, and are particularly relevant in our cohort, as the coal mine fire occurred in a socioeconomically disadvantaged regional area. Individuals with limited access to low-cost primary care or asthma management resources may have experienced poorer symptom control following exposure. Between 2018 and 2021, ED presentation rates for asthma in Australia were consistently highest among individuals in the lowest two socioeconomic quintiles,^41^ a trend also observed internationally.^42^ Regional areas in Australia also had more ED presentations for asthma than metropolitan areas,^41^ which may reflect reduced access to affordable and/or state-funded general practitioners and respiratory specialists.

Apart from wheeze, other common asthma symptoms—such as chest tightness, cough, and shortness of breath—showed varying associations with mine fire-related PM_2.5_ exposure. Symptoms of chest tightness remained elevated up to 7 years after the mine fire. This may reflect the increased prevalence of wheeze among participants, but it could also be linked to other mechanisms, including COPD and cardiopulmonary diseases such as chronic bronchitis, heart failure, and ischemic heart disease.

Chronic cough was elevated 4 years after the mine fire, particularly in people without prefire asthma, but attenuated to null thereafter. This contrasts with our previous HHS analysis,^19^ which found worsening chronic cough associated with increased PM_2.5_ exposure 8.5–9 years postfire, also predominantly in those without prefire asthma, suggesting the current study may have been underpowered to detect associations in this group. In the full Adult Survey cohort, chronic cough and chronic phlegm were increased in association with mine fire-related PM_2.5_ exposure 2.5 years postfire,^15^ with similar trends observed in the respiratory subcohort, although CIs were wider and included null effects. Increased mucus production can be caused by infection or in response to inhaled foreign particles, and if PM_2.5_ was deposited on the mucociliary escalator, mucus production may have increased to assist in its removal. Chronic cough may be caused by various chronic lung diseases, including interstitial diseases and asthma, and when accompanied by phlegm, it may be in response to mucus clearance. However, since chronic cough increased 4 years postfire but chronic phlegm did not, the increase in chronic cough may indicate heightened airway responsiveness rather than mucus-related clearance.^43^

The association between both nocturnal and resting dyspnea with mine fire-related PM_2.5_ was evident 2.5 years postfire in our respiratory subcohort, but not in subsequent follow-ups. Both forms of dyspnea may be linked to asthma or other acute cardiopulmonary conditions, such as heart failure or obesity. However, dyspnea generally is a nonspecific symptom with a complex interplay of physiological and psychological factors. This may explain why the relationship between dyspnea and mine fire PM_2.5_ exposure did not follow the same pattern of association observed for wheeze across the survey waves.

Our results suggest there may be residual respiratory health implications for an exposed population after a medium-term smoke event, particularly among people with asthma, but also among those without. A better understanding of these long-term effects can inform targeted protective interventions during future smoke exposure events. Properly fitted N95 masks and air purifiers may prevent escalation of symptoms and hospitalizations, but may be less accessible in regional and lower-resource settings and would need to be prioritized in government resource allocation. Other measures, such as reducing outdoor activity, closing windows and doors, or using community-based clean-air spaces, may help mitigate exposure. Enhanced screening, assessment, and treatment for respiratory conditions in fire-prone areas, particularly in the first 5 years postexposure, would support improved respiratory disease management.

### Strengths and limitations

A major strength of this analysis was that participants were followed up across four time points within a 9-year period. We were able to estimate individual-level mine fire-related exposure, outcomes, and confounders, whereas many studies investigating health-related exposure effects from PM_2.5_ use an ecological design. We also oversampled people with asthma to ensure a vulnerable group was assessed longitudinally for any additional health impacts related to the mine-fire exposure. Potential selection bias arising from differences between those who participated in the respiratory subcohort and invitees who did not participate was addressed by adjusting for covariates that differed by participation status^21^ in the regression analysis.

A limitation of the study was the sample size of 519, which was determined based on a priori power calculations for detecting longitudinal changes in forced expiratory volume.^32^ This may have limited the study’s power to detect exposure-related changes in respiratory symptoms over time, particularly in formal statistical interaction tests. Bias due to loss to follow-up at W3 and W4 was addressed by imputing follow-up outcome data for these participants. Sensitivity analyses suggested this attrition had little impact on the estimated PM_2.5_ exposure effects across the survey waves, supporting the robustness of our findings.

Another source of bias in the analysis could include measurement error associated with self-reporting. For example, data used to estimate individual mine fire-related PM_2.5_ exposure included self-reported time-location diaries, which may have been subject to recall bias, as these were collected 2.5 years postmine fire. The impact on exposure estimates, however, is likely to be nondifferential, potentially biasing estimates towards the null, as participants were not asked to report their own exposure levels but rather to report their locations during the mine fire period, and, in turn, those data were combined with independently modeled smoke distribution.

Finally, given that our cohort was recruited from socioeconomically disadvantaged communities, our findings should be generalized to the broader population with caution. However, they may be applicable to other disadvantaged regional and rural locations, which is particularly important as these areas are vulnerable to the impacts of climate change.

## Conclusions

This study addresses a gap in longitudinal research on the long-term impacts of coal mine fires on respiratory health. While there was some variation over time, these findings suggest that previously observed increases in chest tightness, dyspnea, and chronic cough associated with Hazelwood coal mine fire-related PM_2.5_ were no longer evident. The exception was wheeze with evidence that the effects of PM_2.5_ exposure persisted up to 9 years later, particularly in those with asthma premine fire.

## Conflicts of interest statement

M.J.A. has received investigator-initiated grants from Pfizer, Boehringer-Ingelheim, Sanofi and GSK for unrelated research. He has undertaken an unrelated consultancy for Sanofi and received a speaker’s fee from GSK. The remaining authors declare that they have no conflicts of interest with regard to the content of this report.

## Acknowledgements

*The Respiratory Stream clinics were set up in facilities provided by The Healthcare Center, Morwell, and Central Gippsland Health Service, Sale. We thank Shantelle Allgood, Susan Denny, and David Poland, who oversaw all aspects of participant recruitment, and Sharon Harrison for assistance with purchasing, logistics, and setup of the clinics. We also thank the scientists who conducted testing during assessment waves, Annie Makar, Mikayla Thomas, Thomas McCrabb, Faizel Hartley, Jacqui Kleiner, and Isabella Chicas. Finally, we express our appreciation to all the participants who took part in this study; without whom, this research would not be possible.*

## Supplementary Material
