# Supplementary material for: Most respiratory symptoms have resolved 9 years after PM2.5 exposure from the Hazelwood coal mine fire
Source: Environ Epidemiol. 2025 Dec 30;10(1):e450. doi: 10.1097/EE9.0000000000000450 (PMC12755517; doi:10.1097/EE9.0000000000000450)
Supplement: Supplementary file 1 [file ee9-10-e450-s001.pdf]

Supporting Information

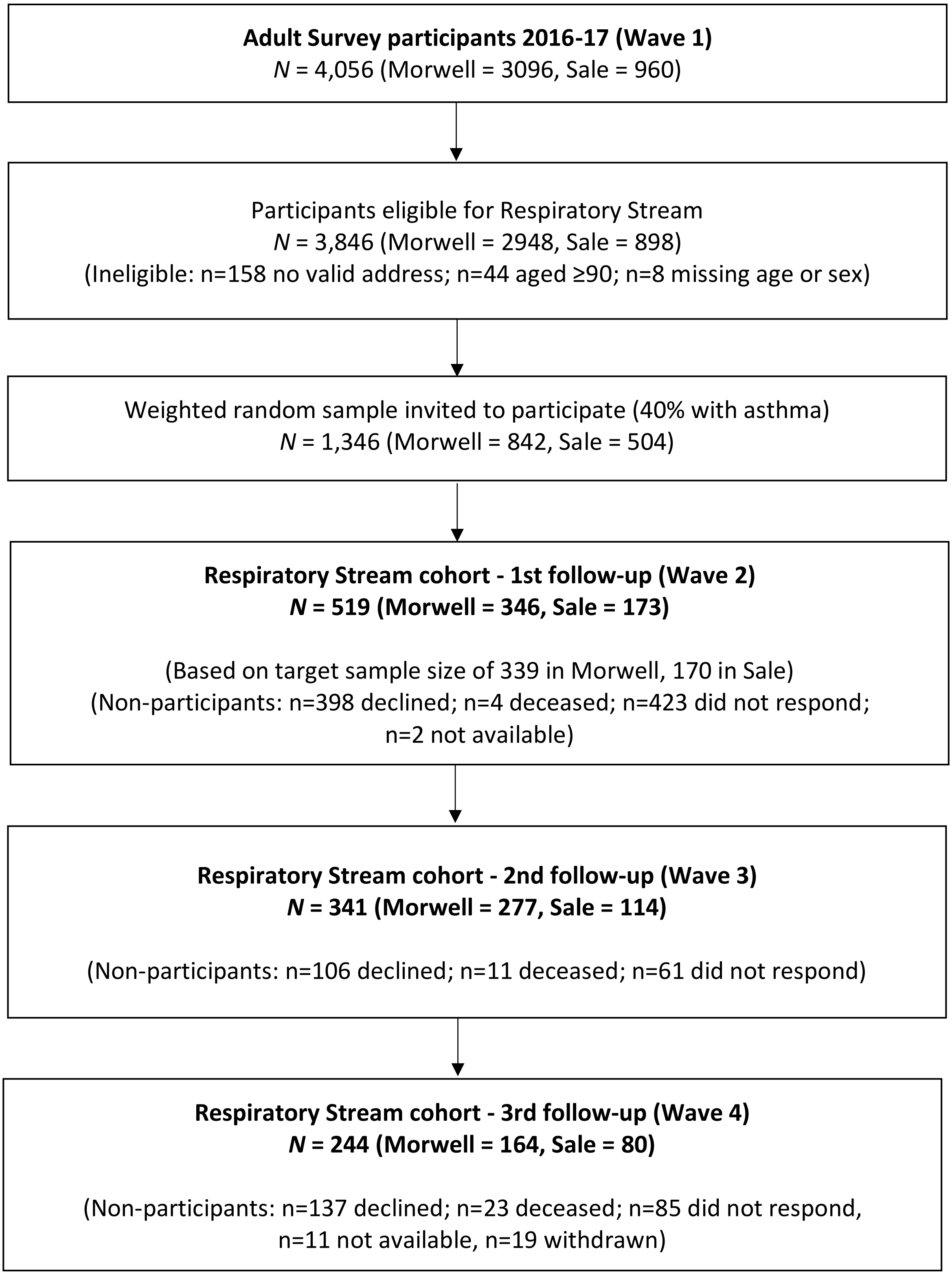

Figure S1. Recruitment into the Respiratory Stream cohort

Table S1. Participant characteristics within exposure groups, in Waves 3 and 4

	Wave 3		Wave 4	
	Morwell (Exposed) N=227	Sale (Unexposed) N=114	Morwell (Exposed) N=164	Sale (Unexposed) N=80
**Daily mean mine fire-related $PM_{2.5}$ μg/m³, median [IQR]**	11.8 [7.2, 17.5]	0.0 [0.0-0.0]	11.5 [7.1, 18.4]	0.0 [0.0-0.0]
**Age in years, median [IQR]**	51 [39, 64]	56 [43, 65]	51 [38, 61]	57 [47, 64]
**Female, n (%)**	195 (57.3%)	111 (64.0%)	93 (56.7%)	51 (63.8%)
**Education, n (%)**				
Up to Year 10	49 (21.9%)	19 (16.8%)	33 (20.2%)	13 (16.2%)
Year 11-12	45 (20.1%)	20 (17.7%)	30 (18.4%)	16 (20.0%)
Post-secondary	130 (58.0%)	74 (65.5%)	100 (61.3%)	51 (63.8%)
**Employment status, n (%)**				
Employed	114 (50.7%)	55 (49.5%)	90 (55.2%)	42 (53.8%)
Other (retired, home, study)	78 (34.7%)	45 (40.5%)	48 (29.4%)	28 (35.9%)
Unemployed/unable to work	33 (14.7%)	11 (9.9%)	25 (15.3%)	8 (10.3%)
**Occupational exposures, n (%)**	82 (36.1%)	39 (34.2%)	62 (37.8%)	28 (35.0%)
**IRSAD score (2011), median [IQR]**	863 [806, 916]	926 [866, 962]	854 [806, 916]	926 [864, 962]
**Self-reported pre-mine fire asthma, n (%)**	110 (48.5%)	45 (39.5%)	85 (51.8%)	32 (40.0%)
**Self-reported pre-mine fire COPD, n (%)**	10 (4.4%)	4 (3.5%)	7 (4.3%)	3 (3.8%)
**Smoking status, n (%)**				
Never-smoker	118 (52.0%)	55 (48.2%)	89 (54.3%)	37 (46.2%)
Ex-smoker	75 (33.0%)	47 (41.2%)	55 (33.5%)	39 (48.8%)
Current smoker	34 (15.0%)	12 (10.5%)	20 (12.2%)	4 (5.0%)
**Cigarette pack years in smokers, median [IQR]**	12.0 [2.5, 22.5]	15.0 [5.3, 28.0]	13.0 [3.6, 23.2]	12.5 [5.0, 25.5]

$PM_{2.5}$, particulate matter <2.5 μm; COPD, chronic obstructive pulmonary disease; IRSAD, Index of Relative Socio-economic Advantage and Disadvantage.
Data displayed as: frequency (%) for categorical variables and median [IQR] for continuous variables. Missing data ranged from 0% to 2.3%.

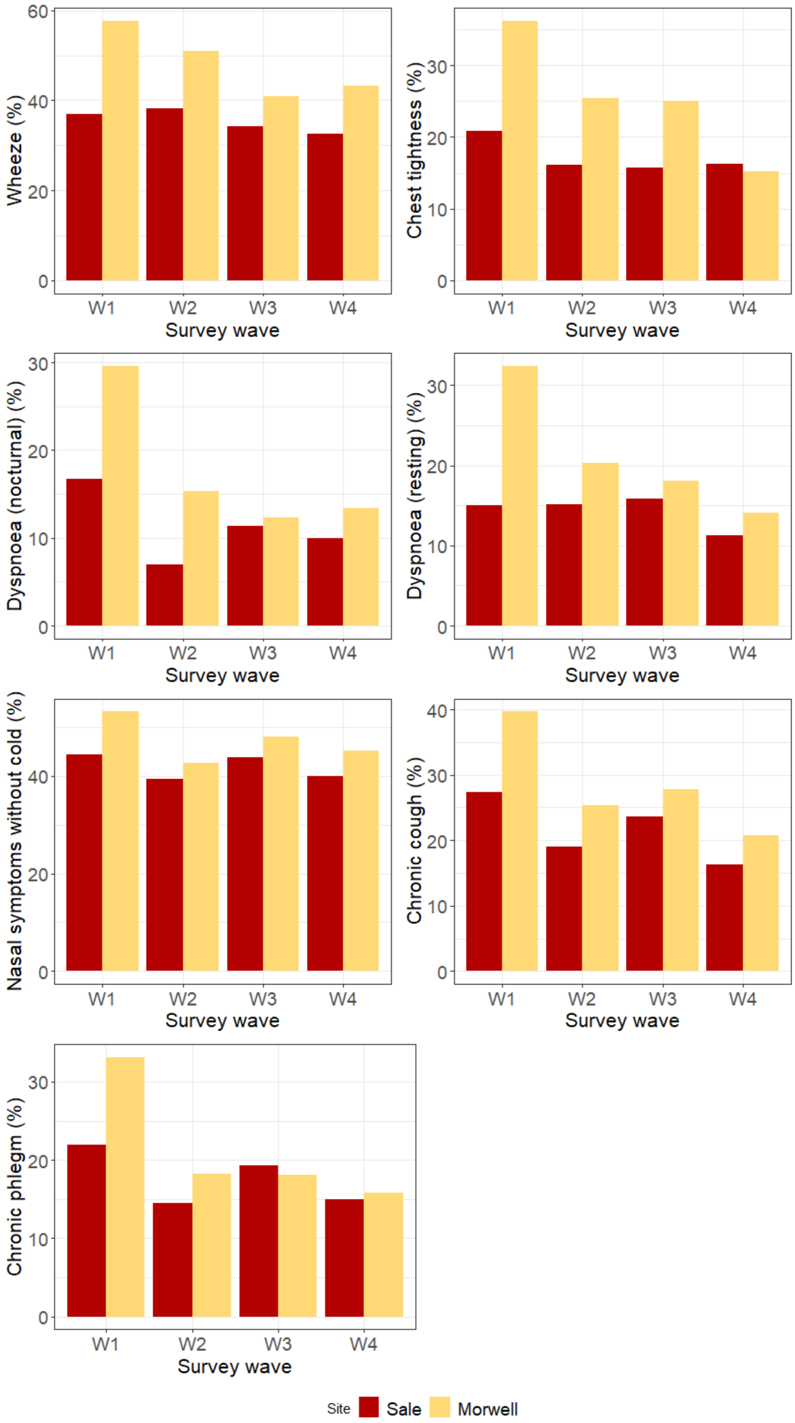

Figure S2. Prevalence of respiratory symptoms by survey wave and study site

Note: Wheeze, chest tightness, dyspnoea (nocturnal), dyspnoea (resting), and nasal symptoms without cold were indicated if experienced in the previous 12 months. Chronic cough and chronic phlegm were indicated if experienced in at least three of the previous 12 months.

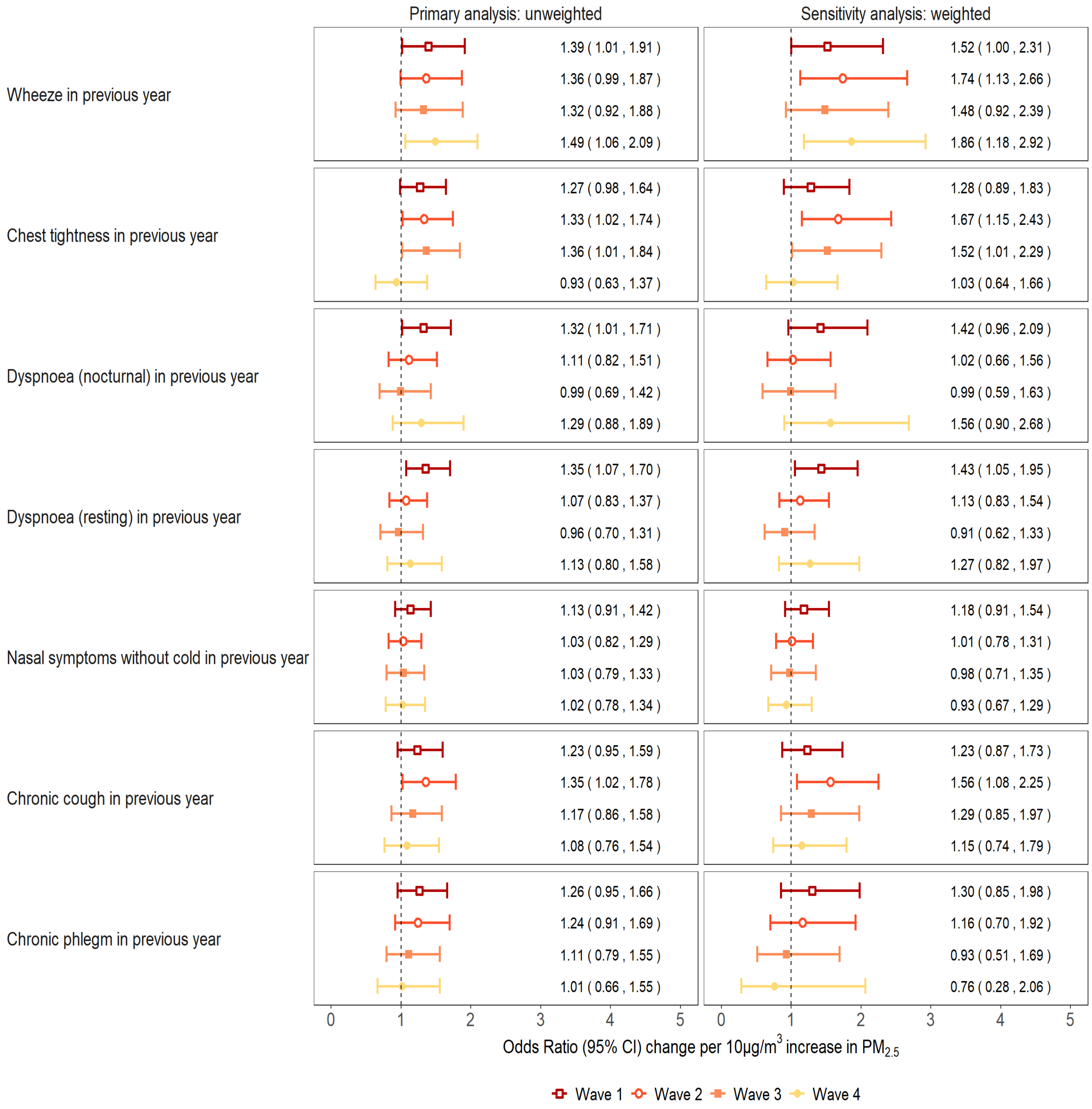

Figure S3. Comparison of primary analysis regression results with regression results weighted for oversampling of asthmatics.

Notes: “Chronic” is defined as at least 3 of the previous 12 months. All models were adjusted for age, sex, employment status, education level, socioeconomic status, smoking, cigarette pack years, occupational exposure, and self-reported asthma or COPD diagnosed pre-mine fire.

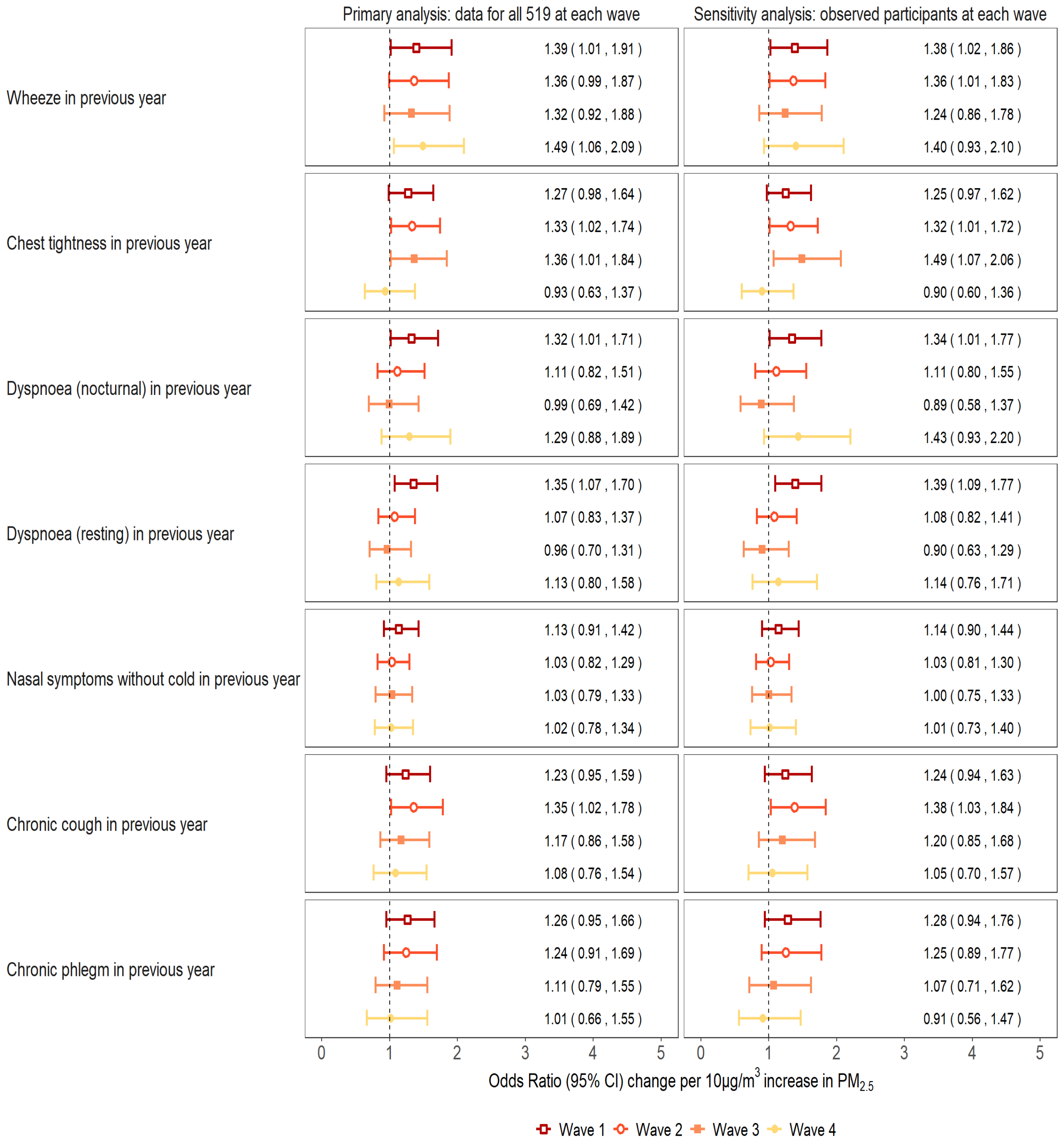

Figure S4. Comparison of primary analysis regression results with regression results based on data including only participants observed at each survey wave

Notes: “Chronic” is defined as at least 3 of the previous 12 months. All models were adjusted for age, sex, employment status, education level, socioeconomic status, smoking, cigarette pack years, occupational exposure, and self-reported asthma or COPD diagnosed pre-mine fire.

Table S2. Comparison of Wave 4 responders to non-responders for participant characteristics and respiratory symptoms across survey waves

	Wave 4 non-responders (N=275)	Wave 4 responders (N=244)	Total (N=519)	P-value
Wave 1 data[3]				
**Age**[1]	54.9 (17.2)	51.4 (14.5)	53.2 (16.1)	0.018
**Female**	162 (58.9%)	144 (59.0%)	306 (59.0%)	p>0.9
**Morwell**	182 (66.2%)	164 (67.2%)	346 (66.7%)	0.852
**Education level**				0.042
Up to Year 10	73 (27.0%)	46 (18.9%)	119 (23.2%)	
Year 11-12	57 (21.1%)	46 (18.9%)	103 (20.1%)	
Post-secondary	140 (51.9%)	151 (62.1%)	291 (56.7%)	
**IRSAD Score for 2011**[2]	866 (818, 926)	877 (818, 945)	868 (818, 927)	0.077
**Mean exposure to fire-related $PM_{2.5}$ ($\mu g/m^3$)**[2]	7.2 (0.0, 14.0)	7.2 (0.0, 13.4)	7.2 (0.0, 14.0)	0.860
**Pre-fire asthma**	115 (42.1%)	117 (48.0%)	232 (44.9%)	0.186
**Smoking status**				0.003
Non-smoker	138 (50.2%)	126 (51.6%)	264 (50.9%)	
Ex-smoker	82 (29.8%)	94 (38.5%)	176 (33.9%)	
Current smoker	55 (20.0%)	24 (9.8%)	79 (15.2%)	
**Wheeze**	134 (48.9%)	129 (52.9%)	263 (50.8%)	0.380
**Chest**	77 (28.1%)	84 (34.4%)	161 (31.1%)	0.129
**Dyspnoea (nocturnal)**	61 (22.3%)	70 (28.7%)	131 (25.3%)	0.105
**Dyspnoea (resting)**	70 (25.6%)	67 (27.6%)	137 (26.6%)	0.690
**Nasal symptoms without cold**	132 (48.4%)	128 (52.5%)	260 (50.3%)	0.379
**Chronic cough**	96 (35.4%)	87 (35.7%)	183 (35.5%)	p>0.9
**Chronic phlegm**	89 (32.5%)	63 (25.9%)	152 (29.4%)	0.122
Wave 2 data[3]				
**Wheeze**	126 (45.8%)	116 (47.5%)	242 (46.6%)	0.725
**Chest**	59 (21.5%)	57 (23.4%)	116 (22.4%)	0.673
**Dyspnoea (nocturnal)**	28 (10.2%)	37 (15.2%)	65 (12.5%)	0.110
**Dyspnoea (resting)**	50 (18.2%)	46 (19.0%)	96 (18.6%)	0.822
**Nasal symptoms without cold**	103 (37.5%)	113 (46.3%)	216 (41.6%)	0.050
**Chronic cough**	57 (20.9%)	63 (25.8%)	120 (23.2%)	0.211
**Chronic phlegm**	45 (16.4%)	43 (17.6%)	88 (17.0%)	0.726
Wave 3 data[3]				
**Wheeze**	41 (33.9%)	91 (41.4%)	132 (38.7%)	0.202
**Chest**	23 (19.0%)	52 (23.6%)	75 (22.0%)	0.342
**Dyspnoea (nocturnal)**	12 (9.9%)	29 (13.2%)	41 (12.0%)	0.487
**Dyspnoea (resting)**	19 (15.7%)	40 (18.2%)	59 (17.3%)	0.654
**Nasal symptoms without cold**	56 (46.3%)	103 (46.8%)	159 (46.6%)	p>0.9
**Chronic cough**	28 (23.1%)	62 (28.2%)	90 (26.4%)	0.369
**Chronic phlegm**	23 (19.0%)	40 (18.2%)	63 (18.5%)	0.884

[1] Mean (SD; [2] Median (IQR); [3] Missing data: n=0 to 6 for Waves 1 and 2 and n=178 for Wave 3 non-respondents (154 in Wave 4 non-responders). Wheeze, chest tightness, dyspnoea, and nasal symptoms were indicated if experienced in the previous 12 months. Chronic cough and chronic phlegm were indicated if experienced in at least three of the previous 12 months.
